# Identification of Umami Taste in Sous-Vide Beef by Chemical Analyses, Equivalent Umami Concentration, and Electronic Tongue System

**DOI:** 10.3390/foods9030251

**Published:** 2020-02-26

**Authors:** Young-Hwa Hwang, Ishamri Ismail, Seon-Tea Joo

**Affiliations:** 1 Institute of Agriculture & Life Science, Gyeongsang National University, Jinju 52828, Korea; philoria@gnu.ac.kr; 2 Division of Applied Life Science (BK21+), Gyeongsang National University, Jinju 52828, Korea; ishamriismail@unisza.edu.my; 3 Faculty of Bioresources and Food Industry, Universiti Sultan Zainal Abidin, Besut Campus, Terengganu 22200, Malaysia

**Keywords:** umami, sensory, sous-vide, electronic tongue, IMP, 5’-nucleotides

## Abstract

Behaviour of umami compounds that are associated with non-volatile compounds on slow cooking regimes remains less explored. This study aims to assess the ability of the electronic tongue system on the umami taste from sous-vide beef *semitendinosus*. The identification was based on the taste-enhancing synergism between umami compounds 5’-nucleotides (IMP, GMP, AMP, inosine, and hypoxanthine) and free amino acids (glutamic and aspartic acid) using the estimation of equivalent umami concentration (EUC) and electronic tongue system. Sous-vide cooked at 60 and 70 °C for 6 and 12 h and cooked using the conventional method at 70 °C for 30 min (as control) were compared. The temperature had a significant effect on 5’-nucleotides, but aspartic and glutamic acid were not influenced by any treatments applied. Sous-vide cooked at 60 °C tended to have higher inosine and hypoxanthine. Meanwhile, desirable 5’-nucleotides IMP, AMP, and GMP were more intensified at the temperature of 70 °C. The principal component analysis predicted a good correlation between EUC and the electronic tongue, with sous-vide at 70 °C for 12 h presenting the most umami. Therefore, the electronic tongue system is a useful tool in food processing, particularly in determining complex sensory properties such as umami, which cannot be evaluated objectively.

## 1. Introduction

Moist heat cooking is the standard cooking method for tough cut meats such as *semitendinosus*, *semimembranosus*, and *biceps femoris* [1]. Sous-vide is one of the examples of moist heat cooking. This cooking method often uses lower cooking temperatures (<95 °C) and longer cooking times as a function to minimize surface drying and to bring advantages to a gastronomic outcome [2,3]. The primary benefit of using sous-vide cooking is that the end-product can be precisely and robustly controlled by selecting suitable cooking conditions (i.e., temperature and time).

From our previous study on tough cuts of beef *semitendinosus* [4], we noticed prolonged cooking at 60 and 70 °C produces lower shear stress, with sous-vide cooked at 60 °C presenting better water retention and color properties. After this finding, curiosity about the palatability of meat led to the exploration of a taste study. Roldán et al. [5] observed that flavor from sous-vide lamb was affected by the temperature and time of cooking. They found that lamb cooked at low or moderate cooking conditions (60 °C for 6 and 24 h or at 80 °C for 6 h) is more likely to have lipid-derived flavor compounds. Meanwhile, at moderately high temperature for long times (80 °C for 24 h) promoted a desirable meaty flavor due to the formation of volatile compounds from Strecker degradation. Besides, previous literature has also evidenced that sous-vide cooking preserves sensory attributes, which minimize the loss of volatile aromatic compounds, resulting in the palatability of cooked meat [5,6,7]. However, information regarding umami taste and non-volatile compounds is still limited.

Umami is an essential sensory feature of meat and plays a significant role in palatability. The umami taste is unique with savory, brothy, or beefy characterizing this broad taste. Several numbers of substances impart the umami sensation, predominantly amino acid glutamate and 5’-nucleotides (mainly inosine-5’-monophosphate (IMP) and guanosine-5’-monophosphate (GMP)) [8]. Also, adenosine-5’-monophosphate (AMP) and aspartic acid, to a lesser extent, contribute to the umami attribute [9]. However, the taste of umami is not clear as compared to sweet and salty because it is formed from the chemical reaction between substances. Although umami is the typical taste of monosodium glutamate (MSG) [10], the MSG taste itself is not very palatable [11]. Yamaguchi and Ninomiya [11] found that the apparent umami taste appears when IMP and glutamate are present in the food system, likely due to the synergistic between both substances. 

The mechanical instrument to measure texture properties are common in meat quality but unusual for sensory measurement. Currently, the sensory features also can be measured using an automated sensing machine, namely an electronic tongue. The taste sensor can measure specific tastes objectively and uniformly [12]. Electronic tongue ability is not only limited to evaluate saltiness, sourness, bitterness, and sweetness but also complex tastes such as umami and astringent. Moreover, this taste sensor is highly correlated to human sensory scores, and it even offers satisfying taste results [13]. Therefore, the objective of this study is to examine the ability of an electronic tongue on the umami attributes at different cooking temperatures (60 and 70 °C) and times (6 and 12 h) of beef *semitendinosus* as a comparison to the conventional cooking method at 70 °C for 30 min. The factors that contribute to umami, such as 5’-nucleotides and free amino acids, and their synergism effect are also investigated.

## 2. Materials and Methods 

### 2.1. Raw Materials and Sampling

Ten *semitendinosus* muscles from Hanwoo beef were purchased from a local meat processing plant at 36 h postmortem (25–30 months old; quality grade 3). The fresh weight of the beef muscles was 1803 ± 550 g. The experiment was randomly divided into 6 and 12 h groups, with each group allocated five muscles (*n* = 5). Before heat treatment, the beef was cut into 2.5 cm thick slices (weight 195–211 g), with the distal and proximal ends discarded. Only two slices were taken randomly from each muscle and assigned as treatments 60 and 70 °C. This procedure was applied to both groups of 6 and 12 h. Then, the muscle was placed in an individual vacuum package, and they were kept refrigerated at 4 °C overnight. 

At 48 h postmortem, the vacuum-packed beef was cooked at 60 and 70 °C for 6 and 12 h using a Sous Vide Travellortech Precision Cooker Immersion Circulator (Travellortech, Ilion, NY, USA). For the conventional cooking method (labeled as a control), the five slices from different muscles were chosen randomly from groups 6 and 12 h (three slices from the 6 h group and two slices from the 12 h group; *n* = 5). Subsequently, the muscles were packed without vacuum inside a polypropylene plastic packaging and cooked at 70 °C for 30 min using a water bath (JSWB-22T, 30 L, Gongju, South Korea). Since the meat in a conventional cooking method such as braising is subjected directly to the boiling water, it often produces imbalance outcomes such as flavorless dry meat and flavorful released juice. Thus, in the current study, we modified the method of conventional braising (control treatment) by cooking the beef in a package at a well-done temperature (70 °C) and limited the cooking time (30 min) as a function to avoid vigorous effects on the meat. This minimizes the loss of flavor inside a bag and preserves the taste compounds of meat. The temperature of water in the bath for sous-vide and conventional cooking was monitored using thermocouples (Testo, model 735–2, Lenzkirch, Germany). Immediately after cooking, the packed-meat was transferred into icy cold water for one hour and then kept under refrigeration at 4 °C overnight before analysis.

### 2.2. 5’-Nucleotides Measurement

The measurement of 5’-nucleotides were carried out by high-performance liquid chromatography (HPLC) based on method of Jayasena et al. [14]. Then, 5’-nucleotides: adenosine-5’-monophosphate (AMP), inosine-5’-monophosphate (IMP), guanosine-5’-monophosphate (GMP), inosine, and hypoxanthine were determined. Five grams of cooked meat was minced and homogenized using a high-speed homogenizer (IKA, model T25D, Staufen, Germany), with cold 20 ml 0.5 M perchloric acid at 10,000 rpm for 15 s. Homogenate was centrifuged at 3000× *g* at 4 °C for 10 min and the supernatant was filtered using filter paper No. 1 (Whatman Ltd., Little Chalfont, UK). After filtration, the residuum again was mixed and vortexed with 10 mL 0.5 M perchloric acid and transferred into the same tube by filtration with filter paper No. 1. The clear filtrate was adjusted to pH 6.0 with cold 5 N potassium hydroxide (KOH) and centrifuged at 3000× *g* at 4 °C for 10 min to allow the salt precipitate to the bottom of the tube. After centrifugation, the pH-adjusted supernatant was filtered with filter paper No. 4 (Whatman Ltd., Little Chalfont, UK) and brought to a volume of 50 mL by adding cold 0.5 M perchloric acid (pH 6.0 adjusted with 5 N KOH). The supernatant was filtered through a 0.45 μm polytetrafluoroethylene syringe filter (SF13-HLB45-M, Futecs Co. Ltd., Daejeon, South Korea) into a 2-mL wide opening screw-top glass HPLC vial (PN: 5182-0557, Agilent, Santa Clara, CA, USA).

The filtrate was analyzed by a Hewlett-Packard HPLC system (Model 1100, Germany) using UV detection at 260 nm. Separation of the 5’-nucleotides was achieved on a C18 column (4.6 × 250 mm, 5 μm particle size, Acclaim ^TM^ 120, Thermo Scientific, Waltham, MA, USA) with the temperature of the column set at 25 °C. Mobile phase A was 0.06 M dipotassium hydrogen phosphate and 0.04 M potassium dihydrogen phosphate at pH 7.0. Mobile phase B was a mixture of HPLC-grade water and methanol (20:80 *v*/*v*). The separation was performed in a gradient elution mode with a constant flow rate at 0.8 ml/min, stop time at 16 min, and post time at 5 min. The gradient program is shown in Table 1. The injection (10 μL) with a needle wash program was used after each injection, using a washing mixture of water and methanol (1:1 *v*/*v*). Identification of 5’-nucleotides was carried out by comparing the retention time in samples with their retention time in standard (AMP, IMP, GMP, inosine, and hypoxanthine; Sigma-Aldrich Co., St. Louis, MO, USA). The concentrations were quantified based on the calibration curve and data was expressed as g/100 g of sample.

### 2.3. Glutamic and Aspartic Acid Determination

Glutamic and aspartic acid were analyzed and identified using HPLC based on the method of Mæhre et al. [15]. Free amino acids were prepared with 0.4 g of minced meat and hydrolyzed under nitrogen gas at 110 °C for 16 h with 15 mL of 6 M HCL. The aliquots (100 μL) was re-dissolved with Mili-Q water (1 mL) after evaporating to dryness with nitrogen gas. Then, they were filtered through a 0.45-μm polytetrafluoroethylene syringe filter. The standard solution and hydrolyzed samples were derivatized with O-pthaldialdehyde (OPA) (PN: 5061-3335, Agilent, Santa Clara, CA, USA) after mixing with borate buffer at pH 10.4 using an autosampler. The autosampler injector program is shown in Table 2. Standard/sample solution was injected through an Agilent ZORBAX Eclipse AAA column (4.6 × 150 mm, 5 μm particle size, Agilent, USA) at 338 nm and column compartment temperature was set at 40 °C (left and right). Mobile phase A contained 40 mm NaH_2_PO_4_ pH 7.8 (adjusted with 10 N NaOH), and mobile phase B contained a mixture of acetonitrile, methanol, and water (45:45:10 *v*/*v*/*v*). The separation was performed using a gradient elution program, as shown in Table 3, with a flow rate at 1.5 mL/min, stop time at 25 min, and post time at 5 min. The individual peaks of glutamic and aspartic acid were identified based on the retention times of standard compounds.

### 2.4. Equivalent Umami Concentration (EUC)

The estimation of umami can be measured based on the concentration of monosodium glutamate (MSG) by EUC value (mg MSG/g), which is equivalent to the umami intensity. The synergy effect between umami amino acids and 5’-nucleotides is represented by the following equation [16]:

(1) $$Y=\;\sum a_{i}b_{i}\;+\;12.18(\sum a_{i}b_{i})\left(\sum a_{j}b_{j}\right)$$

where *Y* is the EUC (mg MSG/g); *a_i_* is the concentration (mg/g) of each umami amino acid (aspartic or glutamic acid); *a_j_* is the concentration (mg/g) of each umami 5’-nucleotide (IMP, GMP, or AMP); *b_i_* is the relative umami concentration (RUC) for each umami amino acid to MSG (glutamic acid: 1 and aspartic acid: 0.077); *b_j_* is the RUC for each umami 5’-nucleotide to IMP (IMP: 1, GMP: 2.3, and AMP: 0.18); and 12.18 is a synergistic constant based on the concentration of mg/g used.

### 2.5. Umami Determination by Electronic Tongue

The electronic tongue system (INSENT SA402B, Tokyo, Japan) was used to determine sous-vide taste information. The instrument included sensor arrays (reference and lipid/membrane electrodes), an autosampler, an electronic unit for data acquisition, and a chemometric data analysis software package. This system was composed of five taste sensors, and each sensor was fixed with a unique artificial lipid. The sensors were named as AAE (to detect umami substances), CA0 (to detect sourness substances), C00 (to detect bitter substances), AE1 (to detect astringent substances), and CT0 (to detect saltiness substances). The inner solution consisting of 3.33 M potassium chloride in saturated silver chloride solution was provided by INSENT Inc. (Atsugi-shi, Japan) and used for the sensor arrays of lipid/membrane and reference electrode [13]. All sensor was preconditioned in a standard solution as described by Woertz et al. [17], and a sensor check was done routinely before every measurement to ensure the system works properly. 

The preparation of the water-soluble extraction was based on the method of Hwang et al. [18]. Minced meat (50 g) was stirred in 200 mL hot distilled water at 95°C (1:4 *w*/*v*) for 10 min before centrifugation at 3000× *g* for 10 min. The supernatant was then filtered through filter paper No. 1 (Whatman Ltd., Little Chalfont, UK) prior to analysis by the electronic tongue.

**Figure 1:**
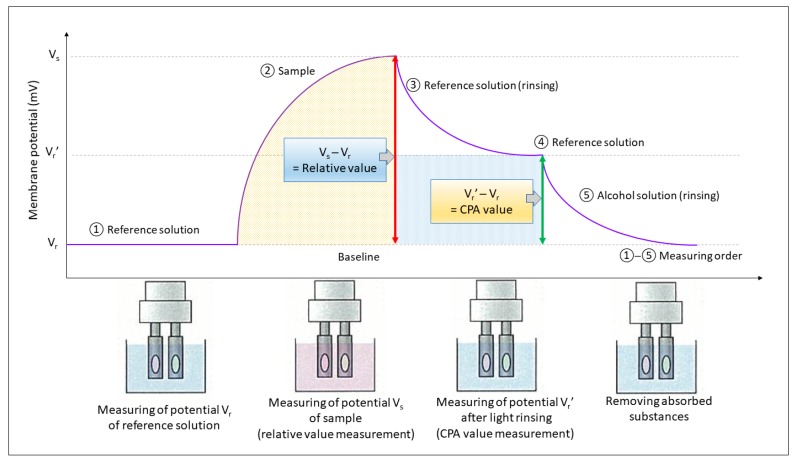
Measurement diagram. The relative value is the initial taste at sensory evaluation, including sourness, saltiness, and umami. The CPA value is the adsorption of bitter and astringent substances. Note: the reference solution should be tasteless or at low concentration than the tested sample. Adopted from Kobayashi, Habara, Ikezazki, Chen, Naito, and Toko [13].

The measurement procedure was as follows: first, the sensor arrays were immersed in the reference solution (inner solution) to obtain the membrane potential, V_r_. Second, the sensor arrays were immersed in the sample solution to obtain the membrane potential, V_s_. Third, the sensor arrays were rinsed lightly with the reference solution and, subsequently, they were immersed again in the reference solution to obtain the potential, V_r_’. Finally, the sensor arrays were rinsed with an alcohol solution (100 mm KCl, 10 mm KOH, 30% ethanol) to remove adsorbed substances before measuring the next sample. The relative value and CPA value (change of membrane potential caused by adsorption) are shown in Figure 1. The sequential measurement was repeated four times for each sample, with the first measurement was discarded to enable conditioning of the sensors. Only the umami response output was taken and presented in this paper. 

### 2.6. Data Analysis

The results of umami related compounds and umami taste evaluation were recorded as mean and standard error of mean (SEM). All assays were performed in triplicate. The interaction between temperatures and times (2 temperatures × 2 times) were performed by analysis of variance general linear model (GLM) using SPSS version 23 (IBM Corp., SPSS Inc., Chicago, IL, USA). The multiple comparisons between control and sous-vide treatments were done at the 5% level by Tukey’s test. Principal component analysis (PCA) was used to test the discrimination performances between cooked samples and tested variables. The first two components (F1/F2) were used to develop the variable and observation plot, and the correlation was observed from the two-dimensional map. PCA was run using XLSTAT software (Addinsoft INC, Brooklyn, NY, USA).

## 3. Results and Discussion

### 3.1. 5’-Nucleotides

The concentration of five 5’-nucleotides were analyzed in the sous-vide cooked meat. As presented in Table 4, all 5’-nucleotides were significantly affected by temperature, while the effect of time was only not significant on the IMP and hypoxanthine (*p* = 0.061 and 0.968, respectively). IMP was comprised of higher composition than the other 5’-nucleotides and it was expected to contribute mainly to the umami threshold [16,19,20]. Besides IMP, other 5’-nucleotides such as GMP and AMP were also important components in imparting umami taste [21]. In all treatments, beef sous-vide cooked for 12 h at 70 °C showed significantly higher IMP, GMP, and AMP, with a GMP value double of that of 6 h of cooking at the same temperature. Unexpectedly, sous-vide cooked at 60 °C, regardless of cooking time, recorded a lower concentration of IMP, GMP, and AMP, but instead, it led to significantly higher contents of inosine and hypoxanthine. The data presented here are similar to those reported for cooked meat samples in previous literature for pork [9], goat [22], and beef [23], which found lower IMP and higher inosine at 60 °C, although there are some differences.

As shown in Table 4, the content of IMP of control was decreased by at least 6% after 30 min of cooking (initial IMP = 49.63 g/100 g), while the content of IMP for sous-vides decreased by 20%–28% and 38–39% after prolonged cooking at 70 and 60 °C, respectively. The degradation of IMP evidenced the significant effect of temperature was more prominent and can be elaborated further by either two mechanisms: (i) dephosphorylation of IMP into inosine; or (ii) IMP and ribose as flavor precursors involve in a number of secondary reactions to yield heterocyclic volatiles compounds [24,25]. This could be explained by which reaction takes place depending on the temperature applied [24]. The reason why 60 °C cooking has lower IMP values and 70 °C cooking has higher IMP values could be elaborated by enzymatic activity, as suggested by Tomioka et al. [26]. In our previous experiment, we investigated the proteolytic activity between temperatures and times of sous-vide cooked beef *semitendinosus*, and we found that at 60 °C cooking had higher proteolytic activity than at 70 °C. This enzyme activity did not change even after 12 h of cooking period [4]. Similarly, Ishiwatari, Fukuoka, Hamada-Sato, and Sakai [23] studied the kinetic model of cooked meat and they found that cooked meat heated above than 60 °C could slow down the activity of the enzyme. They also found that complete deactivation of the IMP-decomposition enzyme activity was reached at 64.1 °C.

### 3.2. Aspartic Acid and Glutamic Acid

Among all the amino acids, aspartic and glutamic acid play a primary role in contributing to umami taste [27]. Therefore, in this paper, we focused only on these amino acids rather than profiling of all amino acids in the sous-vide cooked meat. Interestingly, both aspartic and glutamic acids did not have statistically significant differences at any temperature and time of sous-vide treatment. When viewed at initial values (aspartic acid = 0.49 g/100 g and glutamic acid = 0.59 g/100 g), all the sous-vide treatments contributed to an increase in amino acids of aspartic and glutamic. The present data were in tandem to the finding by Rotola-Pukkila, Pihlajaviita, Kaimainen, and Hopia [9], who also found no difference in both amino acids at different temperatures and times on pork loins. According to Yamaguchi and Ninomiya [11], the detection of glutamate was noticeably lowered in the presence of IMP, likely due to the synergistic effect. However, the results of glutamic acid presented in Table 4 do not seem to be affected by IMP compounds.

### 3.3. Equivalent Umami Concentration (EUC)

Synergistic effects measurement has been carried out to determine the taste synergism between umami amino acids and umami 5’-nucleotides [16], as shown in Table 5. From EUC values, it can be clearly seen that the synergistic effect between umami compounds was intensified at 70 °C, with prolonged cooking contributing the most substantial effect even though it has no significance to control. Sous-vide cooked at 60 °C was less influenced by umami compounds and led to a lower degree of EUC values, regardless of cooking time. This implies that the temperature plays a central role in determining the umami taste. However, the EUC’s actual effect on umami taste properties seems to be limited because the presence of other compounds might also contribute to the umami taste. EUC also does not completely represent the umami taste in a complex matrix. Therefore, a further affirmation of the umami taste between sous-vide treatments by using an electronic tongue was carried out.

### 3.4. Electronic Tongue

The electronic tongue is inspired by the biological system from the human tongue. The sense of taste from the human tongue is mediated by taste receptor cells, where a bundle of sensors in the form of 10,000 taste buds are located [12]. Then, taste perception is processed in the brain. Similarly, the electronic tongue system uses sensor arrays to identify specific taste, which can translate the chemical substances of food into an analytical signal. The results are then further analyzed through a chemometric technique to gain final information of the tested samples. In this study, the reason for using the electronic tongue rather than being examined by sensory panelists is due to the electronic tongue sensors being able to be calibrated in order to be reliably consistent [12], so as to measure specific taste objectively. However, this objective cannot be perceived by the human sensory system in which panelists (in this case, untrained panelists) sometimes confused unintelligible words (e.g., umami). Other than safety reasons, the electronic tongue can also provide different access to the sensory evaluation system. 

In the present study, the umami taste attribute of different sous-vide cooking temperatures and times can be seen in Table 5. Umami taste was significantly affected by temperature (*p* < 0.001), cooking time (*p* < 0.001), and by the interaction between temperature and time (*p* = 0.007). There are no significant differences in the umami taste among all the tested samples, except sous-vide cooked at 70 °C for 12 h cooking time. Empirically, the temperature at 70 °C, regardless of cooking time, showed higher umami intensity than at 60 °C. These results are in tandem with the EUC result obtained, as discussed above. Indirectly, this result gives a clear view that umami taste is better at high temperatures. Although taste is a key issue for sous-vide cooking, only limited studies have highlighted the umami, for instance, in pork [9,28] and beef [23]. However, those studies were not directly assessing the sensory parameter by any means but only based upon chemical compounds (e.g., glutamic acid, IMP, and GMP) that represent umami taste. Thus, further argumentation of umami by sensory evaluation of sous-vide at different temperatures and times become difficult due to limited or no related information.

### 3.5. Principal Component Analysis

**Figure 2:**
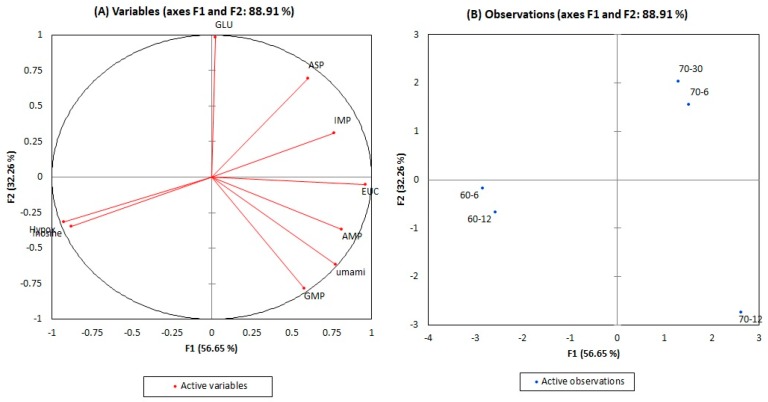
Principal component analysis of umami amino acids and 5’-nucleotides on umami sensory feature in cooked beef *semitendinosus* ((**A**): variables plot; (**B**): observation plot). Abbreviation in the A graph: EUC: equivalent umami concentration; GLU: glutamic acid; ASP: aspartic acid; AMP: adenosine-5’-monophosphate; IMP: inosine-5’-monophosphate; GMP: guanosine-5’-monophosphate; hypox: hypoxanthine. In the B graph, the samples are identified as follows: 60-6 and 60-12: sous-vide cooked at 60 °C for 6 and 12 h; 70-6 and 70-12: sous-vide cooked at 70 °C for 6 and 12 h; 70-30: control, cooked at 70 °C for 30 min.

In order to better visualize the contribution of umami amino acids and umami 5’-nucleotides on the umami sensory feature, discriminative principal component analysis (PCA) was carried out (Figure 2). The PCA variables plot (A) and the observation plot (B) projected in the factor space axes-F1 (56.65%) and axes-F2 (32.26%) were well distinguished. In the variables plot, EUC was located in the middle of axes-F2 and it showed a good correlation to umami (*r* = 0.738). Nevertheless, EUC scores are just an estimation based on the formula umami amino acids and 5’-nucleotides to represent the synergistic fashion of glutamic acid, aspartic acid, GMP, IMP, and AMP, but it does not show true umami attribute. The EUC score shows a better connection to 5-nucleotides IMP (*r* = 0.871), AMP (*r* = 0.679), and GMP (*r* = 0.605) than free amino acid aspartic (*r* = 0.445) and glutamic acid (*r* = -0.060). Conversely, electronic tongue umami represented that only AMP (*r* = 0.912) and GMP (*r* = 0.912) were highly correlated, but weakly correlated to IMP (*r* = 0.333). This unexpected result was supported by the observation of Yamaguchi and Ninomiya [11], who found that the taste intensity of IMP itself is weak, but a strong umami taste is regulated with a glutamate presence. They described this as the synergistic effect between both compounds to produce the umami sensation. We observed separately the EUC score between IMP and glutamic acid (excluding aspartic acid, GMP, and AMP), but our result did not fit with the synergistic effect as suggested, because the umami score for sous-vide cooking at 70 °C for 12 h (data not shown) was shifted to a lower value than control. The score plot (B) in Figure 2 presents a clear difference in the relative position of samples according to their cooking conditions. Thus, sous-vide cooked for 12 h at 70 °C was placed at the lower right quadrant that attributed strong umami. 

In our study, glutamic acid was stable throughout the cooking, as shown in Table 4. It had no difference to control or even to uncooked meat (0.59 g/100g). When it comes to sous-vide cooking, a thing that comes into question is the concentration of IMP not being consistent through cooking. Besides the effect of temperature, as discussed earlier in the text, the effect of cooking time also affects the inconsistency of IMP content although it was not significant (*p* = 0.061; Table 4). Our result was also similar to Ishiwatari, Fukuoka, Hamada-Sato, and Sakai [23] in beef and Sasaki, Motoyama, and Mitsumoto [28] in pork, who found lower IMP with cooking time. As suggested by many researchers, IMP is an important compound in amplifying the umami of glutamate [16,19,20,29,30]. However, it is a very crucial occasion to reach umami by synergism between glutamic acid and IMP in cooked meat because umami for both parties is dependent on the optimal concentration of IMP. Yamaguchi and Ninomiya [11] also described a similar finding where subjective taste intensity was induced to the lower value if the IMP proportion was present at too high or too low concentrations to glutamate. Therefore, further study is necessary for clarifying the two complexes of IMP and glutamic acid for their synergistic effect, at which point of temperature and time can optimally trigger the umami. 

## 4. Conclusions

The umami taste attribute has been examined based on the estimation method of EUC using an electronic tongue system. It was shown that in this study that the temperature effect (60 and 70 °C) is much important than the time effect (30 min, 6 h, and 12 h). The most interesting point to be highlighted is the umami taste could be induced by sous-vide cooking at 70 °C, with the most apparent attribute achieved after prolonged cooking, affirmed by both EUC and the electronic tongue and their strong correlations. Furthermore, correlation by PCA best-described umami with 5’-nucleotides than free amino acids. Therefore, an electronic tongue is the best alternative tool to replace conventional sensory assessment in food processing applications, especially in determining complex taste attributes such as umami.

## Figures and Tables

**Table 1:** 5’-nucleotides gradient elution conditions.

Time (min)	Mobile Phase B (%)
0	2
5	4
7	4
8	30
9	0
16	0

**Table 2:** Injector program.

Line	Function	Amount	Reagent	Vial Position
1	Draw	2.5 μL	Borate buffer pH 10.4	1
2	Draw	0.5 μL	Sample	11
3	Mix	3 μl in air, max speed 2×		
4	Wait	0.5 min		
5	Draw	0 μL	HPLC-grade water (for needle was using water in uncapped vial)	2
6	Draw	0.5 μL	O-pthaldialdehyde (OPA)	3
7	Mix	3.5 μL in air, max speed 6×		
8	Draw	0 μL	HPLC water (for needle was using water in uncapped vial)	2
9	Mix	4 μL in air, max speed 6×		
10	Draw	0 μL	Acetonitrile	5
11	Draw	32 μL	HPLC-grade water	4
12	Mix	18 mL in air, max speed 2×		
13	Inject			

**Table 3:** Free amino acids gradient elution conditions.

Time (min)	Mobile Phase B (%)
0	2
0.5	2
20	57
20.1	100
23.5	100
23.6	2
25	0

**Table 4:** 5’-nucleotides (AMP, IMP, GMP, inosine, and hypoxanthine, g/100 g) and free amino acids (glutamic and aspartic acid, g/100 g) of beef *semitendinosus* heated at different temperatures and times.

Temperature	Control	60 °C		70 °C		SEM	*P* Temp (T)	*P* Time (t)	*P* T*t
Time		6 h	12 h	6 h	12 h				
AMP	0.66 ^b^	0.53 ^b^	0.61 ^b^	1.25 ^a^	1.44 ^a^	0.08	<0.001	<0.001	0.542
IMP	46.73 ^a^	30.94 ^b^	30.21 ^b^	35.84 ^ab^	39.71 ^ab^	2.95	0.024	0.061	0.443
GMP	5.41 ^b^	4.22 ^b^	6.98 ^b^	6.04 ^b^	12.38 ^a^	0.89	0.001	<0.001	0.059
Inosine	1.32 ^c^	2.90 ^b^	3.95 ^a^	1.45 ^c^	1.74 ^c^	0.14	<0.001	<0.001	0.012
Hypoxanthine	0.85 ^c^	1.24 ^a^	1.13 ^ab^	0.82 ^c^	0.90 ^bc^	0.07	<0.001	0.968	0.188
Glutamic Acid	0.66	0.62	0.61	0.66	0.59	0.03	0.899	0.172	0.125
Aspartic Acid	0.57	0.54	0.54	0.60	0.55	0.03	0.261	0.768	0.379

^a–c^ Different superscript letters within the same row mean significantly different between treatments (*p* < 0.05). SEM: standard error of mean (*n* = 5). AMP: adenosine-5’-monophosphate; IMP: inosine-5’-monophosphate; GMP: guanosine-5’-monophosphate.

**Table 5:** EUC (mg MSG/g) and umami taste (mV) of beef *semitendinosus* heated at different temperatures and times.

Temperature	Control	60 °C		70 °C		SEM	*P* Temp (T)	*P* Time (t)	*P* T*t
Time		6 h	12 h	6 h	12 h				
EUC	505.03 ^a^	330.84 ^b^	346.34 ^b^	444.05 ^ab^	532.92 ^a^	32.98	<0.001	0.286	0.279
*Umami	2.40 ^b^	2.36 ^b^	2.35 ^b^	2.49 ^b^	2.73 ^a^	0.04	<0.001	<0.001	0.007

^a–b^ Different superscript letters within the same row mean significantly different between treatments (*p* < 0.05). SEM: standard error of mean (*n* = 5). * Umami is referred to as the umami taste measured by the electronic tongue system. EUC: equivalent umami concentration.
